# Bridging Perspectives: Exploring the Convergence of Clinimetrics and Network Theory in Mental Health Assessment and Conceptualization

**DOI:** 10.3390/jcm13061814

**Published:** 2024-03-21

**Authors:** Elena Tomba, Giuliano Tomei

**Affiliations:** 1 Department of Psychology, University of Bologna, 40127 Bologna, Italy; giuliano.tomei2@unibo.it; 2 Department of General Psychology, University of Padua, 35131 Padua, Italy

**Keywords:** clinimetrics, clinimetric assessment, network theory, network analysis, literature review, clinical assessment, psychiatric disorders, psychopathology

## Abstract

**Background**: Clinimetrics and network analysis are two methodological approaches that, despite different origins, share the goal of improving mental health clinical assessment beyond the limitations of classical psychometrics. Despite their common goal and comparable conceptualization of clinical assessment, the potential connection and integration between these approaches has not been explored. The aim of this review is to identify meeting points for the potential integration of clinimetrics and network theory. **Methods**: A literature review was conducted by examining key works in clinimetrics and network theory and comparing similar concepts from the two approaches. **Results**: Two main areas of theoretical and methodological convergence and complementarity between clinimetrics and network theory were identified, as follows: the characteristics of clinical indexes and the strategies to assess and organize complex clinical data. These topics encompassed sub-topics related to the influence of individual symptoms on clinical presentation, longitudinal assessment of conditions, influence of comorbidities, and standardized procedures for case formulation. **Conclusions**: Results provide an indication of the potential for integration for these approaches in a single, clinically oriented methodology for psychological and psychiatric illness conceptualization and assessment. Despite the literature search strategy limitations, the results provide a basis for further exploring the potential for developing an integrated methodology for clinical assessment and treatment planning.

## 1. Introduction

Assessing and identifying phenomena in the field of mental health is a complex and challenging task [1], primarily due to the absence of specific biomarkers or pathognomonic signs [1,2]. Moreover, mental health conditions are primarily diagnosed based on clinical observations and reported symptoms, as well as being affected by huge variability between individual cases. These hurdles underscore the importance of promoting a comprehensive approach to the assessment and classification of mental health symptomatology and conditions. Thus, psychometrics was developed to apply mathematical modeling to the measurement of psychological variables which cannot be directly measured, like intelligence, mood, or personality; this was based on the assumption of the presence of a latent variable that is responsible for the observable characteristics. Latent variable models are statistical models applied in psychiatry and psychology to explain the connections between observable symptoms and unobservable constructs [3,4]. Despite its significant contribution in developing an evidence-based approach to study mental health constructs, psychometrics presented weaknesses, mostly in assessing phenomena in a clinical setting; it was developed independently from the clinical setting and, as such, its core assumptions find limited validity and applicability in such contexts [5]. As discussed by Fava and colleagues [6], this detachment between psychometrics and clinical experience caused two main problems affecting the validity of measuring instruments in the field of mental health, as follows: the idea that all items (or symptoms) of a psychometric questionnaire carry equal importance [6,7] and the problems surrounding the lack of interrelatedness between comorbid conditions [8].

Similar difficulties in translating statistical models to the reality of clinical medicine had already been noted. Within the field, a remarkable attempt to integrate data obtained from statistical methods with information from clinical medical experience was made by Alvan R. Feinstein, who in 1982 introduced the term “clinimetrics” to indicate the domain of measurements concerning medical indexes, rating scales, and other expressions used to describe or assess physical symptoms, signs, or other manifestations of illnesses [9]. The original intent of Feinstein was to overcome the roadblock faced by clinical medicine at that time [10], to expand the medical taxonomy of his period to also encompass clinical experiences, and to start considering those relevant clinical phenomena that should be a core part of any clinical assessment or research in medicine. These included, among others, the patient’s own experience of illness, their available social resources, and the disorder’s progression rate [11]. Thus, Feinstein proposed to consider additional indexes along the ones normally included when conducting a medical assessment, to differentiate between patients that would otherwise be considered similar by virtue of sharing the same medical diagnosis [11]. Remarkably, this was nothing new for clinicians who, except for using the word itself, have been communicating using clinimetric indexes all along [11]. The progression of illness, overall severity of the medical disorder and functioning impairment, available social support, and response to previous treatments are all aspects that are regularly factored in by clinicians, despite the absence of formal methods to capture this information [12]. Through clinimetrics, Feinstein attempted to bring back the process of clinical assessment within the realm of clinical practice, stepping back from delegating the assessment process to scientists from nonclinical domains [13]. Feinstein stressed the importance of developing “new approaches […] of clinical investigation that can augment the scientific basis of clinical practice, while rehumanizing the contents of research data and restoring analytic emphasis to the art of patient care” [10]. Examples of clinimetric indexes commonly used in clinical medicine are the Jones criteria for rheumatic fever [14], the New York Heart Association Functional Classification [15], and Apgar’s method of scoring the newborn’s condition [16].

Several researchers, with Fava [6] at the forefront, followed by Bech [17] and Emmelkamp [18], highlighted the limitations of the psychometric model in the field of mental health and promoted a conceptual revision of clinical assessments to include clinimetrics principles. Their work integrated psychometrics with clinimetric principles to pave the way for significant advancements in the clinical assessment processes in psychology and psychiatry, by promoting revisions in three major areas, (1) the introduction of new criteria to develop and choose clinical measures [16,19]; (2) the introduction of new areas and methods of clinical assessment [13,19]; and (3) the conceptualization of new approaches to clinical reasoning to improve the standardization of clinical data collection, such as macro- and micro-analysis [17,18]. While clinimetrics has been enthusiastically adopted by some, others have raised concerns about this model. Streiner [20,21] argued that clinimetrics is not an innovation as much as a redundant and ultimately unnecessary reinterpretation of the psychometric model. Emmelkamp himself, a key figure in the field of clinimetrics, while acknowledging the benefits of the clinimetric approach (such as the focus on a scale’s sensitivity to change), warns against abandoning classic psychometrics before providing definitive proof of the clinimetric approach’s effectiveness [18].

In the second decade of the 2000s, a group of researchers from statistical and research methodology backgrounds, independently from the clinimetric approach, also addressed the limitations of classical psychometrics in measuring mental health symptoms. Fried [22], in particular, raised concerns about the Latent Variable model, characterizing most available psychometric screening instruments and psychiatric taxonomy, in which all symptoms are considered roughly independent and equally relevant to a specific disorder, because all result from the same underlying condition. Such an assumption, according to Fried [22], is hardly tenable as it contradicts real-life clinical observations, where symptoms interact and vary in importance. This observation followed Borsboom’s initial critique of the assumption of the local independence of symptoms [23], in which he observed that diagnostic criteria do often contradict the axiom necessary for the Latent Variable model to be valid, by indicating direct functional relations between signs and symptoms. This position appears, in general, to be aligned with what Fava and colleagues observed in previous works [6].

An alternative approach to the Latent Variable Model was proposed, initially called network perspective [23,24,25] and later known as the network theory of mental disorders [26,27,28]. Network theory is built upon the foundations of the wider network approach, which has a long history in mathematics and physics [28] and which focuses on the identification of system components and their relationships. At its core theory, the network theory of mental disorders postulates that mental states act as networks, where individual entities (from psychopathological symptoms to social or medical variables) are represented as nodes of the network. These nodes are connected by edges, which represent the probabilistic dependency between two nodes after conditioning on all other variables in the data [26,28]. However, in psychology, we currently do not have objective, definitive, and empirically established theories on how symptoms influence each other; it is, therefore, impossible to estimate the structure of a psychological network based on theory alone. Such a structure must, therefore, be inferred from the data, posing the question of how to extract it. This question eventually led to the conception of psychometric network analysis [28], an ensemble of statistical methods that allow us to determine the structure and interactions between the components of a network by estimating dependencies between variables, while conditioning on other variables [27]. Each psychological network is, thus, conceived as a complex system that persists in a state of equilibrium; external factors (life events, biological triggers, and prolonged exposure to stress) can activate a node, triggering a cascade effect of the activation of other nodes. The propagation of this cascade depends on the strength of the edges involved; stronger edges will carry the perturbation further. This model represents a significant step away from the Latent Model approach, which postulates that psychiatric symptoms are the manifestation (and the only measurable part) of a relatively small set of underlying psychiatric disorders causing those symptoms, similarly to the classical disease paradigm of Western medicine [29]. The disease model advanced by network theory instead postulates that symptoms do not cluster because of a shared underlying disorder that they originate from (the Latent Variable)—they cluster because they can cause, and maintain, each other [22]. In the last decade, network psychometrics has received ever increasing attention from clinicians and researchers and, since its inception, the number of published studies using network psychometrics studies in different psychology specialties has been growing steadily [30], though the sweeping enthusiasm for networks raised some questions about the methodological robustness of the studies [31,32]. Notwithstanding these limits, network psychometrics has already established itself as a powerful method to analyze psychological data from a variety of clinical conditions [30,33], such as depression [34,35], eating disorders [35,36], anxiety [35], psychosis [35,37,38,39], and PTSD [40,41,42].

Both clinimetrics and network theory therefore move away from traditional psychiatric diagnostic taxonomy and psychometric measurements, in order to establish a more comprehensive theoretical and practical framework for understanding, measuring, and capturing the dynamic nature of mental health conditions as they present in everyday clinical practice. Despite the different starting points—clinical experience for clinimetrics [11] and measurement methodology for network theory [26]—the two approaches seem to converge on many of their core concepts. While there are other relevant theoretical conceptualizations of mental health conditions which provide as relevant a framework as clinimetric and network theory and have been somewhat juxtaposed to the latter [43], to provide a comprehensive overview of the various theoretical frameworks would be past the scope of this work.

As such, the goal of this manuscript is, therefore, to bring together clinimetrics and network theory, to discuss their common theoretical assumptions and methodological applications, and to promote a measurement methodology effective for case formulation and treatment planning.

## 2. Materials and Methods

The following work consists of a theoretical literature review according to the classification of reviews and associated methodologies from Paré and colleagues [44]. Seminal published works in the fields of clinimetrics and network theory were analyzed to identify the areas of possible convergence between these two approaches. In particular, online databases (Pubmed, PsycInfo) were searched for reviews, using *psychological network*, *network psychometrics*, *network analysis*, and *psychological network analysis* for psychological network theory, as well as clinimetric* for clinimetrics. During title screening, all articles that did not relate to psychology, clinical psychology, or psychiatry, or that did not discuss the components of the theoretical model were excluded. Similarly, all articles not in English were excluded during the initial selection. A further analysis of the abstracts of the remaining studies led to the inclusion of six published articles, which were individually analyzed by the authors, as follows: for clinimetrics, the works of Fava and colleagues on the application of clinimetrics to clinical psychology and psychiatry [12,45,46] were analyzed by one of the authors (E.T.) to identify relevant topics within the clinimetrics literature and the manual retrieval of the associated literature. Simultaneously and independently, the seminal works of Borsboom and Cramer [47] and the more recent advancements in the network theory [48,49] were reviewed by the other author (G.T.) to identify relevant topics within the network theory literature and to support the manual retrieval of the associated literature. The two authors (E.T. and G.T.) then proceeded to independently identify which practical and conceptual analogies could be found between the network psychometrics theory and practice and clinimetrics. In the case of disagreement between the two authors, consensus was reached through multiple rounds of full-text revision and discussions with the aid of a research assistant (E.S.) when necessary. Once the main areas of convergence between clinimetrics and network theory were identified, one of the authors (E.T.) proceeded to manually retrieve published works within clinimetrics pertaining to the relevant topics, while the other author (G.T.) simultaneously and independently manually retrieved the relevant published works within network theory pertaining to the same relevant topics.

## 3. Results

**Figure 1:**
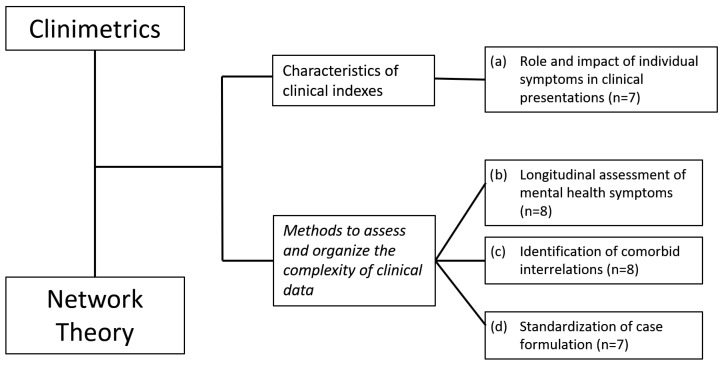
Flowchart of clinimetrics and network theory comparison conceptualization and specific analogous topics identified.

Extraction and comparison of theoretical principles and methodological guidelines between clinimetrics and network theory highlighted two main shared topics, the characteristics of clinical indexes [9,28] and the methods to assess and organize the complexity of clinical data [25,45]. Within these two macro-groups, four specific areas of common ground were identified. Encompassed within the characteristics of clinical indexes group there is (a) the role and impact of individual symptoms in clinical presentations [9,12,17,28,46,50,51]. Within the methods to assess and organize the complexity of clinical data group, the following topics were identified: (b) the longitudinal assessment of mental health symptoms [25,45,52,53,54,55,56,57], (c) the identification of comorbid interrelations [8,11,12,23,24,26,58,59], and (d) the standardization of case formulation [12,13,18,49,60,61,62]. See Figure 1 for a flowchart of the article selection procedure.

### 3.1. Characteristics of Clinical Indexes

#### (a) Role and Impact of Individual Symptoms in Clinical Presentations

Both clinimetrics and network theory emphasize the importance of evaluating the specific role and impact of individual symptoms in a disorder’s presentation, which provide valuable information for case formulation and treatment planning. In clinical practice, the number of symptoms a patient reports do not necessarily mirror the severity of their clinical presentation. The intensity and quality of each symptom and the patient-perceived impairment must also be considered. Identifying the most impairing symptoms, as well as understanding their origin, triggers, and maintenance dynamics are all critical components of the clinical assessment and case formulation process [46]. Below, the individual contributions and meeting points between clinimetrics and network theory for improving the evaluation of the specific role and impact of individual symptoms in clinical presentations are reported [9,12,17,28,46,50,51].

One of the goals of clinimetrics is to move past the idea of severity as a result of the number of co-occurring symptoms. Clinimetrics proposes a more complex approach, where the significance of symptoms might be influenced by their quality and intensity rather than their mere presence. However, items from classic psychometric questionnaires tend to have the same weight [9,12,46], meaning that from a clinical point of view all items (or symptoms) virtually exert the same impact on the individual. Consequently, the total score of a psychometric scale built on such premises will identify the relevant characteristics of given presentation; as an example, it would be impossible to determine if a high score in a scale for assessing depressive symptoms is the result of many mild depressive symptoms or few, extremely severe manifestations [9]. This critical limitation is also shared by psychiatric nosography at large, which, in most cases, equals severity of a disorder to the number of diagnostic criteria present, rather than the intensity or quality of these symptoms [9].

Clinimetrics prompts clinicians to develop and choose valid and reliable measures able to recognize both “floor” symptoms that only appear in severe cases, as well as “ceiling” symptoms that are present across mild and severe presentations [17]. Questionnaires that assign the same importance to items representing floor and ceiling symptoms fail to capture this clinically meaningful difference and should be avoided or, when adopted in clinical practice, appropriately interpreted [17]. The appraisal of individual symptoms during clinical assessment may also vary depending on how they are weighted by the clinician. Another proposal stemming from clinimetrics is, therefore, to adopt a clinical assessment approach in which the significance of symptoms might be interpreted in light of their relationship with other symptoms, suggesting their reciprocal should be carefully taken into account during a clinical evaluation [12].

Clinimetrics also encourages researchers and clinicians to develop and adopt measures with limited internal redundancy. Ordinarily, psychometric scales include several items or subscales investigating similar constructs, under the misguided assumption that this approach will ensure minimal loss of information [9]. Talking about a psychometric measure and using Bech’s own words “each individual item’s degree of independent information should be examined […]. To allow each symptom to express its particular piece of information (its particular prevalence) corresponding to the area it covers on the ruler, there must not be much overlap between the symptoms” [17].

Network theory proposes a similar theoretical approach to that of clinimetrics, focusing on the importance of evaluating the specific role and impact of individual symptoms in the clinical presentation of a disorder.

Network theory emphasizes the importance of a node’s centrality and the weight of its edges within a network as an index of the real impact of that specific symptom in the clinical presentation. Essentially, the centrality of a node represents its importance, meaning the capacity of that node/symptom to affect other nodes/symptoms of a network [50]. A node exerts its effect over the network through its edges connecting with other nodes; the more numerous and strong connections a node presents, the higher its capacity to alter the network structure or activate other nodes [62]. The absolute sum of the weights of a node’s edges is called strength centrality [28] and it is the most commonly reported centrality metric in psychological networks [28]. Considering the weight of an edge represents the strength of the conditional association between nodes [28], strength centrality provides important information on how much the perturbation of a specific node will affect the surrounding nodes; the higher the edge weight, the more the effect will be transmitted to the rest of the network.

Network theory, like clinimetrics, also emphasizes avoiding redundancy in psychological measures. Flake and Fried [51] highlighted the need to avoid measuring the same construct multiple times in network models, as it can lead to distortions in the network structure and bias the measurement of a node’s centrality.

### 3.2. Methods to Assess and Organize Complex Clinical Information

#### 3.2.1. (b) Longitudinal Assessment of Mental Health Symptoms

Both clinimetrics and network theory emphasize the importance of longitudinally assessing symptoms of psychological conditions. Both approaches postulate that the contextualization of a presentation within a temporal framework, encompassing the progression of symptoms’ severity, sequence of symptoms’ onset, and the influence of specific symptoms in subsequent clinical presentation, are critical aspects for selecting the appropriate treatment [30,45,49,53]. In the following paragraph, clinimetrics’ and network theory’s proposals to improve longitudinal clinical assessment are presented [25,45,52,53,54,55,56,57].

Feinstein’s model of clinimetric assessment proposed the concept of “transfer stations”, crucial temporal points in treatment where the clinician reassesses the patient’s stage of illness and plans treatment accordingly [45,52]. In the 1990s, Fava and Kellner extended the staging model to also include psychiatric disorders, adapting it from its original use in clinical medicine [45]. The staging model aims to determine the current stage of a psychiatric disorder, ranging from prodromal to chronic or recovered stages. By focusing on the extent and timing of disease progression at a particular point in time, the staging model helps to identify patients along the continuum of illness development and to differentiate between early and milder clinical manifestations, as well as features of progression and chronicity [53]. By considering the symptomatology within a longitudinal framework, the staging model helps clinicians understand the progression of a disorder at a particular point in time, as well as a patient’s current location on the continuum of the course of illness. The current state of the illness represents an alternative assessment method to the traditional cross-sectional psychiatric nosography [45]. The staging model of psychiatric disorders has been applied to a wide range of psychiatric conditions [54], staging models for levels of treatment resistance, and for loss of therapeutic effects during continuation or maintenance treatment [56]. However, the clinimetrics proposal of a staging model derives from clinical experience and observation and there is currently no definitive empirical evidence supporting these models.

Network psychometrics also emphasizes the significance of disease dynamics and temporal progression [25], as in Feinstein’s clinimetric conceptualization of transfer stations [52]. According to network theory, networks are dynamic entities where their state at a given time is influenced by their previous state. This interdependence is captured through equations that describe how the previous state affects the current state or how variables interact with each other over time along a trajectory determined by the initial connections between variables [25]. While network theory does not identify specific stages of illness, it does allow their development through a specific statistical methodology that can retrieve temporal networks, networks which are built from longitudinal data and include directed edges [57]. Temporal networks enable a more detailed analysis of the long-term effects of individual symptoms on the clinical presentation. These networks offer insight into which symptoms are associated with an increase in other symptoms at a future time point, as well as the magnitude of these predictive relationships. This provides access to the identification of key symptoms driving the worsening of psychiatric conditions or triggering comorbidities, as well as unlocking the understanding of underlying dynamics governing shifts from health to illness and vice versa [55]. Importantly, temporal networks have been applied to longitudinal data from a single individual (“*n* = 1 time series”), allowing the construction of highly individualized network models where individual-specific dynamics between symptoms can be observed [57]. Temporal networks also allow the estimation of causality between symptoms, where the characteristics of a specific symptom/node help in predicting the future states of other nodes, as well as of the whole network.

#### 3.2.2. (c) Identification of Comorbid Interrelations

A critical element that heavily influences the heterogeneity of clinical presentations in psychiatry is comorbidity. Both network theory and clinimetrics acknowledge the limitations of traditional evaluation processes and the advantage of introducing complementary methods that may be better equipped to capture the complexity and heterogeneity of co-occurring psychiatric conditions [8,11,12,23,24,26,58,59].

Comorbidity, which can be defined as the contemporaneous presence of two or more mental health conditions [63], has been an ever-increasing presence in psychiatric practice. Clinimetrics, however, proposes a different definition for comorbidity [11]. Feinstein defined comorbidities as “any distinct additional clinical entity that has existed or that may occur during the clinical course of a disease that is under study [8]”, which clinicians need to investigate and contextualize within individual clinical presentations. With true comorbidity, Feinstein [8] identified the co-presence of two clearly separate diseases, either contemporaneously present due to a common aetiological cause, or due to one disorder causing the other [58]. Conversely, with spurious comorbidity, Feinstein [8] described a blurred clinical picture where the relationship between two diagnoses may not reflect a true relationship between the disorders; this misclassification could be determined by phenotypic heterogeneity or due to the construction of artificial categories that share the same underlying dimensions or create a discrete boundary where none actually exists, such as in the psychiatric nosological approach [58] (see Table 1). Feinstein’s conceptualization of comorbid entities emphasizes their clinical relevance. Thus, the concept of comorbidity goes beyond just the co-occurrence of disorders and focuses on a more detailed examination of how the co-existing symptoms interact and impact the clinical picture. The complex nature of psychiatric comorbidities and the challenges in their identification call for a standardized procedure that will help the clinician in identifying and separating the various clusters of symptoms based on how they present in clinical reality, rather than following predetermined categories. Fava and colleagues [12] built upon Feinstein’s work, expanding the process of comorbidity assessment to include subsyndromal symptoms, illness behavior, functional capacity, and psychological well-being. Furthermore, Fava, Tossani, and colleagues highlighted various key aspects of clinimetric comorbidity, such as the need to identify appropriate statistical methods to reduce discrepancy between comorbidities across studies, the need to develop and test causal models of comorbidity, and the application of the clinimetric method to move past the disorder-based comorbidity concept by including clinically significant subthreshold conditions and other psychological factors that may influence the choice of treatment [59].

This granular level of the detection of comorbidities is also present in network theory by its focus on the complex connections and dynamics between individual symptoms or edges of the network [24]. In network models, as reported by Cramer et al. [24], nodes tend to cluster together, forming highly connected communities. These communities are usually only weakly connected to nodes from other clusters. However, nodes from different clusters may present strong connections between each other, not unlike nodes within the same cluster [26]. In clinical terms, symptoms from different disorders may be exhibited as strongly related symptoms from the same disorder, leading to comorbidity by reciprocal influence. Nodes that connect clusters in this way are called bridge nodes; comorbidity arises as a node triggers the activation of nodes from neighboring cluster(s) connected by bridge nodes. This can cause the simultaneous activation of different groups of symptoms. It is noteworthy that the notion of certain symptoms transmitting “pathological” activation from one group of nodes to another hinges on the premise that symptoms are not interchangeable, which challenges a fundamental tenet of the traditional taxonomy of mental disorders. This model suggests that symptoms are not equivalent and a diagnosis cannot solely rely on the number of symptoms present, but rather on their intensity and their contribution to the overall clinical presentation [23].

#### 3.2.3. (d) Standardization of Case Formulation

Coherent with its complexity, the standardization of the process of case formulation and comorbidity assessment has been a persistent challenge in the fields of psychiatry and clinical psychology [64]. Both clinimetrics and network models acknowledge the critical need to build standardized procedures to develop case formulations and provide their own contribution, as follows: clinimetrics, through the introduction of macro- and micro-analysis, and network analysis through the development of individual-specific network models [12,13,18,49,60,61,62].

Clinimetrics’ macro- and micro-analysis are two intertwined clinical techniques designed to guide clinicians in the assessment process, particularly in relation to comorbidity management and impact of illness [18]. Macro-analysis involves constructing a designed model of the relationships occurring between syndromes and/or problematic areas experienced by the patient. These relationships, far from static, are subject to change and evolution as the treatment proceeds, requiring ongoing evaluations during the course of treatment. Aa a result, treatment targets may vary during the course of therapy [18]. The experienced psychiatrist or clinical psychologist evaluates the patient’s various syndromes and problems to inform decision-making and treatment prioritization in collaboration with the patient [13]. This increases the patient’s motivation to engage with the treatment, as they will feel the clinician is addressing areas they themselves perceive as critical [18]. Macro-analysis should also include those areas of functioning or life events that influence the clinical presentation, even if not strictly related to psychopathology (i.e., presence of stressors, challenging socioeconomic conditions) accordingly with Feinstein’s original definition of comorbidity [8]. As can be seen in Fava et al. [12], the result of this process is a series of interconnected constructs that influence each other through causal relationships, often depicted using topological representation akin to a network. Once the macro aspect has been explored collaboratively by clinician and patient, micro-analysis should be conducted [13]. The micro-analysis process involves a detailed examination of the specific symptoms of the conditions identified during the macro-analysis and their context, such as onset and specific areas of impairment. This process is crucial to understand specific triggers or behaviors associated with the condition and to select appropriate measurement scales. By identifying the unique characteristics of a patient’s illness experience, clinicians can choose the most suitable assessment tool, thereby overcoming the misconception of a “one size fits all” approach to psychiatric assessment [12]. Both macro- and micro-analysis are dynamic processes that require regular updates as treatment progresses and the patient’s presentation evolves. This approach ensures personalized assessment and intervention based on the patient’s priorities, current presentation, and preferences [12].

From a network theory perspective, person-specific temporal networks provide a way to apply network models for improving the reliability and standardization of case formulation [49]. Person-specific networks (PSNs) are network models built from the data collected on a single individual using the experience sampling method (ESM), which assess momentary states during daily life. Person-specific networks identify two types of associations between variables in a patient, as follows: contemporaneous associations (i.e., symptom A is associated with symptom B at the same time point) and temporal associations (symptom A is associated with symptom B at the following time point) [49]. This method allows for the collection of fine-grained data on the individual’s experiences over time, which can be used to construct a network model representing the interactions between various constructs (such as triggers, symptoms, illness behaviors, life events, and psychological well-being) in that individual [49,61]. The objective of person-specific temporal networks development is to help clinicians discriminate between different groups of subjects and to detect changes within the same subjects over time, improving the standardization and replicability of case formulation [49,62]. Important limits have been identified in the application of PSNs to clinical practice, as some aspects may be missed or inadequately captured, due to the qualitative nature of the limitations of reducing the diversity of context to a set of quantitative variables [49]. Furthermore, alternative explanations for the observed associations, such as indirect causation or the influence of unobserved variables, must be considered [49,60].

## 4. Discussion

The psychometric model has been considerate inadequate to reflect a real clinical setting [6]. To compensate this inadequacy, clinimetrics and network theory proposed alternative theoretical and practical frameworks to capture clinical information. To lend further strength and relevance to these approaches and their contribution to the improvement of clinical assessment, the current work sought to establish a common ground between these two methods and to suggest possible areas where they might integrate and complement each other. To this end, the theoretical foundations and methodological applications of both approaches have been reviewed.

Clinimetrics and network theory have emerged as two distinct approaches to improve the clinical assessment process and offer valuable insights into complex psychological phenomena. While different in origin—clinical observation and statistical methodology, respectively—these approaches converge on the necessity of integrating multiple information sources to create a more dynamic and representative system for clinical assessment [5,19,28,65,66,67]. Arguably, this convergence of separate and independent research lines is, in and of itself, an indicator of the issues that affect the translation of the psychometric model to clinical practice. Even more striking, though possibly not surprising to the attentive clinical scientist, is the fact that both approaches independently identify comparable critical areas in traditional psychiatric taxonomy and questionnaires and propose conceptually similar solutions.

From the information presented in this theoretical review, it emerges how both fields share several important similarities, including the focus on understanding and integrating complex dynamic psychological phenomena starting from the relationships occurring between single symptoms and how these are the key elements for an accurate clinical assessment [12,25].

Both clinimetrics and network theory developed starting from the premise that they can influence each other and are what ultimately determines the clinical presentation of a patient. Furthermore, they agree on the necessity to rethink assessment instruments to reduce the redundancy of items and to increase the ability of instruments to detect subtle changes in symptomatology. Indeed, as observed by Boyle [68], major and minor symptoms can and should be differentiated to reflect the heterogeneous presentation of clinical conditions. Statistically, Cattel [69], almost 50 years ago, had already warned against the inclusion of highly correlated items measuring the same psychological construct. Clinimetrics addresses this issue by focusing on the role of individual symptoms and the development of clinical indexes able to appropriately detect them [9], macro- and micro-analyses [13], and careful clinical reasoning on the role of symptoms [66]. Network theory, on the other hand, proposes the development of mathematical models able to detect symptoms’ centrality and their edges [28,70]. The possibility unlocked by network analysis to identify the most relevant symptoms and their existing relationship has relevant implications for building clinimetrically robust scales; theoretically, it could be possible to differentiate and assign specific scoring weight to individual questionnaire items depending on their centrality in the network structure. Taking into account Faravelli’s and Bech’s observations on the importance of individual symptoms within a clinical presentation [7,17], it is possible to appreciate how clinimetrics and network analysis both distance themselves from the classic psychometric model to focus on the role of specific symptoms’ contributions.

Considering the above, it would seem that the development of tools to measure the importance of individual network nodes (representing a given variable) and to avoid redundancy in their measurement has great potential to improve clinical assessment tools in accordance with both clinimetrics and network analysis’ theoretical principles.

Both clinimetrics and network theory move forward from the cross-sectional taxonomy to underline the importance of a longitudinal evaluation of psychiatric symptoms which takes into account the sequencing, trajectory, and personal experience of illness [71,72]. Through the staging model, clinimetrics offers a powerful conceptual framework and practical tool for clinicians to model their intervention on the patient’s current stage of disease [45,65], rather than relying on a one-size-fits-all approach [54]. The staging model of psychiatric disorders has been enthusiastically adopted by the clinical and academic community and adapted for numerous psychiatric conditions, as reported by Cosci and Fava [54]. Similarly, network theory has also been shifting towards the longitudinal analysis of symptom networks, both at a group and individual level, by focusing on the development of temporal network models [48] to capture the causal relationships between symptoms. Temporal networks offer new and exciting possibilities for the identification and study of the clinimetric stages of illness or to develop new clinimetric models, and it seems worth exploring their joint application to develop or improve clinical indexes and assessment. The clinimetric model of staging relies on the clinician’s individual evaluation of the present symptoms and their impact, with its intrinsic limitations, such as relying on the clinician’s experience and the patient’s ability to recall and disclose relevant information [73]. However, these limitations can be compensated by the application of temporal networks, which allow a detailed longitudinal analysis of the symptoms’ reciprocal influence and their crystallization in those stable patterns (the attractor states) that could represent the statistical foundation to support the identification of the clinimetric stages of illness.

Network psychometrics instead introduces the concept of attractor states, which are equilibrium states that networks converge to and which stabilize after a perturbation [25,74]. This formulation well reflects the uneven progress observed during clinical interventions, which often involve a series of improvements and setbacks until a stable state of health with minimal symptoms is achieved [71,75]. The conceptualization of temporal progression and the influence of specific symptoms on future states in network psychometrics may be aligned with the clinimetric concept of staging, which takes into account a patient’s unique characteristics to determine their stage of illness [45].

The concept of comorbidity is another area where clinimetrics and network theory intersect. The traditional approach to comorbidity in psychiatry has been criticized for oversimplifying complex clinical phenomena [24,46,76,77]. In response, clinimetrics and network theory highlight the importance of evaluating the timing and effect of individual symptoms on an individual’s functioning [66,78]. They both move beyond the simplistic conceptualization of co-occurring disorders and capture the clinical relevance of symptoms interacting with each other in a more nuanced manner, akin to Feinstein’s original definition of comorbidity [8]. In this regard, the clinimetric approach and network theory would seem to complement each other. Clinimetrics underscores the significance of measuring symptoms in a comprehensive and nuanced way and leveraging clinical expertise to evaluate how the severity, duration, and impact of symptoms influence the clinical manifestation [6,66]; network theory contributes to this approach by providing the theoretical and methodological tools to study the interrelationships between symptoms through bridge nodes, identifying which symptoms are responsible for connecting otherwise isolated cluster of symptoms and how their influence affects the clinical picture [47,79].

Considering the conceptual common ground between these two approaches and their clinical implications, network models seem a promising approach to expand the methodological toolbox of clinimetrics by expanding our understanding of psychiatric comorbidities.

In terms of standardizing case formulation process clinimetrics’ macro-/micro-analysis and network theory’s PSNs again appear to complement each other. Case conceptualization, which involves identifying antecedents, cognitive, emotional, physiological, and behavioral experiences of patients in problematic situations, serves as the foundation of evidence-based approaches. The goal of case formulation is to develop a patient-specific model of the relationships between emotions, behavior, cognitions, somatic states, and context [80]. Case formulation in clinical practice varies based on the clinician’s training and theoretical orientation and even standardized procedures, such as those used in cognitive behavioral therapy, can be influenced by subjective factors [68], with consequences on the reliability of the process [72,81,82]. Macro- and micro-analysis help to navigate the various aspects of a formulation, such as syndromes, and psychosocial functioning, treatment history (macro-analysis), as well as the dynamics between symptoms (micro-analysis) [12,13]. For macro- and micro-analysis to contribute to the clinical process, the timing and trajectory of the phenomena of interest should be carefully evaluated [12,18]. PSNs can support macro-/micro-analysis by collecting and analyzing fine-grained information about phenomena of interest, providing a standardized representation of their interactions. Although not mature enough for clinical use, PSNs could hypothetically go beyond retrospective information and integrate complex information into a standardized process [49]. Klipstein and colleagues [49] highlighted how PSNs may prove an important tool in guiding collaborative case formulation; however, they also underlined the importance of “keeping the responsibility for developing a working theory of the patient’s pathology with patient and therapist”. Clinimetrics focuses on allowing patient and clinician to take charge of this very same process and can push the boundaries of formulation to include complex information, which is usually excluded. Again, it is possible to appreciate the complementary interplay between clinimetrics and the network approach; clinimetrics offers a clinically grounded theoretical framework to conceptualize the patient’s presentations and identify areas of interest, where network models provide the statistical methodology to organize, analyze, and interpret this information. The contiguity of network theory and macro-/micro-analysis is further strengthened by their shared use of topological representations to illustrate the dynamics between different elements in a patient’s presentation, see [49]. Both approaches start by examining specific details and then assess how these details impact the overall clinical picture, by affecting related symptoms or areas of the patient’s life. They further agree on focusing on specific elements in order to better understand their impact on the overall clinical picture. By revealing the relationships between various constructs such as triggers, symptoms, illness behaviors, life events, and psychological well-being, person-specific networks can provide a powerful approach to statistically formalize the processes of macro- and micro-analysis and for both network models and clinimetrics to inform and improve the standardization and replicability of case formulation.

Despite the encouraging premises reported in this work, some considerations are necessary for the correct interpretation of the information reported. First of all, only two authors partook in the selection, extraction, and reviewing of content prior to drafting the manuscript, with potential implications for its validity. Secondly, this work is a first attempt at establishing a common ground upon which clinimetrics and network theory might operate together for the benefit of clinicians, researchers, and, most importantly, patients. As such, despite the best attempt at being as thorough as possible in drawing comparisons and establishing divergences, the literature inception for this work was not systematic in nature and might not encompass all possible common aspects between the two approaches. While the seminal publications for both clinimetrics and network theory were included, it is possible that not all relevant studies have been considered due to the non-systematic literature inception carried out for this work.

## 5. Conclusions

Clinimetrics and the network theory of mental disorders have both, in their own right, enriched the process of assessing and conceptualizing mental health conditions [5,30]. Even when considering the limits of the present work, in light of their similitude and, perhaps most importantly, their unique elements, it is difficult to argue against the potential of their combined application. The integration of clinimetrics and network analysis could greatly benefit clinical practice, as it would provide practitioners with more comprehensive and sophisticated assessment tools to make informed decisions about diagnosis, treatment, and care [5,72]. For example, a clinimetrics–network analysis hybrid assessment tool could be used to not only measure the presence or absence of symptoms, but also to understand the relationships between different symptoms, risk factors, and underlying psychological mechanisms. This information would be particularly valuable for guiding diagnosis and treatment decisions, as it would allow practitioners to tailor interventions to the unique needs of each patient. Future studies might want to explore this by conducting systematic literature reviews on this topic and by empirically testing clinimetrics indexes through network models, to further explore this novel yet promising direction

## Figures and Tables

**Table 1:** True and spurious comorbidities based on [58].

**True Comorbidity**	A true relationship between two discrete disorders. Can be either the result of two disorders sharing common etiologic factors or a common diathesis, while remaining two distinct entities, or when one disorder causes (or increases the probability of) the other.
**Spurious comorbidity**	An apparent relationship between two diagnoses that does not reflect the true relationship between the two disorders or diseases. It can be the result of different phenotypic expressions of the same disease, leading to apparent comorbidity, or when one disorder is, in fact, a prodrome or an attenuated form of the other disorder, or again when a disorder might be a subcategory nested within the more general category defining another disorder.

## Data Availability

No new data were created or analyzed in this study. Data sharing is not applicable to this article.
